# Response to Letter to the Editor: “First Admission Neutrophil–Lymphocyte Ratio May Indicate Acute Prognosis of Ischemic Stroke”

**DOI:** 10.5041/RMMJ.10457

**Published:** 2021-10-25

**Authors:** Murat Alpua, Bahar Say, Ilknur Yardimci, Ufuk Ergün, Ucler Kisa, Ozlem Doğan Ceylan

**Affiliations:** 1 Department of Neurology, Kirikkale University, Faculty of Medicine, Kirikkale, Turkey; 2 Department of Biochemistry, Kirikkale University, Faculty of Medicine, Kirikkale, Turkey; 3 Department of Medical Biochemistry, Ankara University, Faculty of Medicine, Ankara, Turkey

**Keywords:** Ischemic stroke, lymphocyte, neutrophil

**TO THE EDITOR**

We have carefully read and evaluated the letter written by Drs Mungmunpuntipantip and Wiwanitkit regarding our article published in the July issue of *Rambam Maimonides Medical Journal*.^1^

Neutrophil/lymphocyte ratio (NLR) is an indicator that is calculated using the neutrophil and lymphocyte values in the whole-blood test, and its popularity is increasing day by day. The NLR values are calculated by proportioning the neutrophil count to the lymphocyte count from the whole-blood test results. Furthermore, NLR is generally accepted as an indicator of inflammation.^2^

We found that the authors were concerned about the sensitivity of the NLR assays. However, neutrophil count and lymphocyte count were analyzed using the same device in each case. In this respect, we think that there was no problem in terms of the sensitivity of the analyses, since NLR is calculated by dividing the relative percentage of neutrophils by the relative percentage of lymphocytes.

In addition, many clinical studies have shown that NLR can be used as a biomarker in many diseases.^3^–^6^ Of course, many factors can affect the NLR. These factors should also be taken into account when making a prognostic evaluation.

## Abbreviations

NLR: neutrophil/lymphocyte ratio.
